# Identifying Challenges in Social Functioning Among Women with Schizophrenia

**DOI:** 10.1192/j.eurpsy.2024.1547

**Published:** 2024-08-27

**Authors:** S. Walha, I. Chaari, A. Samet, C. Ben Ghanem, L. Aribi, F. Charfeddine, N. Mseddi, J. Aloulou

**Affiliations:** Psychiatry « B » department, Hedi Chaker University Hospital, Sfax, Tunisia

## Abstract

**Introduction:**

Schizophrenia spectrum disorders profoundly impacts social functioning, affecting interpersonal relationships, work interactions, and self-care. This disorder often leads to cognitive, perceptual, motor, and emotional challenges that result in social withdrawal.

**Objectives:**

The aim of the study is to identify the specific challenges in social functioning faced by women diagnosed with schizophrenia spectrum disorders.

**Methods:**

We conducted a descriptive cross-sectional study among stabilized female patients with schizophrenia or schizoaffective disorder, in the ‘B’ psychiatry department at Hedi Chaker University Hospital in Sfax, Tunisia, from May to June 2023. We collected both sociodemographic and clinical data from the participants. The Social Functioning Scale (SFS) and Global Functioning Scale (EGF) were used to assess social and global functioning, respectively.

**Results:**

Forty-one patients were included: 65.9% had schizophrenia, and 34.2% had schizoaffective disorder. The mean age was 49.19 years, ranging from 17 to 79 years. More than a third (39%) of our patients had significant impairment in global functioning (EGF<50). The average total score on the social functioning scale was 13.65, with a range from 6.29 to 20.29. Additionally, 39% of our patients exhibited low social functioning, and 51.21% had a high withdrawal score. The most impacted domains were leisure (63.41%) and employment (60.97%), followed by interpersonal behavior (58.53%), prosocial activities (48.78%), independence competence (41.46%), and lastly, independence performance (36.85%).

**Conclusions:**

Social skills training is crucial for enabling women with schizophrenia to function well in their environment.

**Disclosure of Interest:**

None Declared

